# A questionnaire-based cross-sectional study on neuropathic pain in patients with cancer in Japan

**DOI:** 10.1093/jjco/hyaf116

**Published:** 2025-08-01

**Authors:** Saori Hashiguchi, Hiroshi Takahashi, Shuhei Yamamoto, Haruhiko Seki, Yaoki Sonohara, Yuko Tanabe

**Affiliations:** Department of Palliative Medicine, St. Marianna University School of Medicine, 2-16-1 Sugao, Miyamae-ku, Kawasaki, Kanagawa 216-8511, Japan; Primary Medical Science Department, Medical Affairs Division, Daiichi Sankyo Co., Ltd., 3-5-1, Nihonbashi-honcho, Chuo-ku, Tokyo 103-8426, Japan; Data Intelligence Department, Global DX, Daiichi Sankyo Co., Ltd., 3-5-1, Nihonbashi-honcho, Chuo-ku, Tokyo 103-8426, Japan; Value & Access Department, INTAGE Healthcare Inc., 13th Floor, Ochanomizu SolaCity, 4-6 Kanda Surugadai, Chiyoda-ku, Tokyo 103-8426, Japan; Value & Access Department, INTAGE Healthcare Inc., 13th Floor, Ochanomizu SolaCity, 4-6 Kanda Surugadai, Chiyoda-ku, Tokyo 103-8426, Japan; Department of Medical Oncology, Toranomon Hospital, 2-2-2 Toranomon, Minato-ku, Tokyo 105-8470, Japan

**Keywords:** cancer, neuropathic pain, questionnaire, S-LANSS

## Abstract

**Background:**

Some patients with cancer experience cancer-related neuropathic pain. This study investigated the prevalence of possible neuropathic pain in patients with cancer.

**Methods:**

This observational, cross-sectional, questionnaire-based study recruited adult participants with stage ≥II cancer in Japan between June and November 2024. The primary endpoint was the proportion of participants with suspected neuropathic pain (Self-Reported Leeds Assessment of Neuropathic Symptoms and Signs pain scale [S-LANSS] score ≥ 12 points). Secondary endpoints included pain scores (11-point numeric rating scale [NRS]), communication with healthcare professionals, impact on daily activities, and quality of life (QoL) per the EQ-5D-5L.

**Results:**

Responses from 713 participants were analyzed. Of those, 230 participants (32.3%) reported cancer-related pain and 53 (7.4%) had suspected neuropathic pain. Among participants with suspected neuropathic pain, the mean ± standard deviation (SD) pain intensity (NRS) was 5.1 ± 2.7. Over one-third of participants (37.7% [20/53]) with suspected neuropathic pain reported that no healthcare provider had asked about cancer pain before they sought a consultation, 60.4% (32/53) wished their healthcare provider had noticed their pain sooner, 69.8% (37/53) could no longer perform some daily activities due to cancer pain, and 98.1% (52/53) thought their pain needed to be adequately treated for them to live their life going forward. QoL was lower among participants with suspected neuropathic pain versus the overall population (EQ-5D-L mean ± SD: 0.6065 ± 0.2518 vs 0.8204 ± 0.1788).

**Conclusions:**

These findings suggest that pain assessment earlier in the cancer treatment process than standard may improve pain management for patients with cancer.

## Teaser

Among study participants with stage ≥II cancer, 32.3% had cancer pain and 7.4% had suspected neuropathic pain. Patients may benefit from pain assessments in the early stages of cancer treatment.

## Introduction

Important advances in cancer therapy have extended survival times for patients with cancer; however, pain remains a common cancer-related issue [1]. Cancer-related pain can be caused by the primary cancer, metastases, and/or treatments such as chemotherapy, surgery, and radiation therapy. A systematic review found a high prevalence of cancer-related pain, reporting that 39% of patients experienced pain after curative treatment, 55% experienced pain during their cancer treatment, and 66% experienced pain with advanced, metastatic, or terminal disease [2].

Neuropathic pain is a major type of cancer-related pain. It has both physical and psychological impacts, negatively affecting activities of daily living and quality of life (QoL) [3, 4]. The prevalence of cancer-related neuropathic pain varies by cancer type, stage, and treatment. Cancer-related neuropathic pain in the general population of adult patients with cancer has been reported to have a prevalence as high as 44% [5]. Chemotherapy-induced neuropathic pain can lead to chemotherapy dose reduction or discontinuation [6], resulting in poor treatment outcomes.

Cancer-related neuropathic pain is difficult to treat, and only a limited number of analgesic options outside of opioids are currently available [7–14]. Opioid analgesics are effective for nociceptive pain but have a relatively low impact on neuropathic pain [15]. Many patients with cancer who are receiving chemotherapy hesitate to consult with their attending physician about pain and other side effects, as they worry this might distract physicians from treating their cancer [16]. These issues highlight the importance of conducting a comprehensive survey of the neuropathic pain that patients with cancer experience to understand the treatments used and their side effects and to inform the development of an optimal management plan for each patient. The objective of this study was to investigate the prevalence of possible neuropathic pain in patients with cancer, along with pain severity, treatment for pain, patient satisfaction, and QoL.

## Patients and methods

### Study design

This was an observational, cross-sectional questionnaire-based study conducted in Japan that recruited patients between June 2024 and November 2024. Physicians who were members of PLAMED Inc. (Tokyo, Japan) were asked to participate in the study, and those who agreed (hereafter referred to as the attending physicians) recruited patients under their care to the study. The number of survey requests sent to each physician was adjusted by the type(s) of cancer they treated to avoid biasing toward certain cancer types.

Attending physicians received sealed study packets that included an instruction letter, a survey form, and a return envelope. The study questionnaire included questions about the participants’ background and demographic characteristics, pain, analgesic use, and QoL. If respondents experienced pain in more than one location, they were instructed to respond about pain in the single most painful location. The study questionnaire, translated into English, is available in the Supplementary data.

Study packets were given to all eligible patients seen by the attending physician (i.e. they were not specifically selected by the physician), up to a limit of 30 patients. Patients who chose to participate in the study filled out the study questionnaire and returned it to INTAGE Healthcare Inc. (Tokyo, Japan), along with confirmation of their consent to participate. INTAGE Healthcare Inc. handled the study questionnaires and collated the responses. After completion, the questionnaire was sealed, and the contents were not visible to the attending physician.

The study protocol was approved by the Ethics Review Board at TOUKEIKAI Kitamachi Clinic. The study was conducted in accordance with local and institutional ethical standards, the 1964 Declaration of Helsinki and its later amendments, the Japanese Ethical Guidelines for Medical and Health Research Involving Human Subjects [17], the Japanese Act on the Protection of Personal Information, and the revised Personal Information Protection Act. The study was registered in the University Hospital Medical Information Network clinical trials registry (UMIN000054421). All participants provided informed consent to participate in the study prior to responding to the study questionnaire.

### Study participants

Individuals were eligible for inclusion in the study if they had been diagnosed with cancer (stage II or higher), were aged ≥18 years at the time of consent, and were able to understand the study procedures and respond appropriately to the study questionnaire without assistance. There were no prespecified exclusion criteria. Survey eligibility was limited to patients with stage ≥II cancer to ensure that a sufficient proportion of participants with suspected neuropathic pain were included in the study, as patients with advanced cancer tend to have a higher prevalence of cancer pain compared with nonadvanced cancer [18].

### Study endpoints

The primary endpoint was the proportion of participants with suspected neuropathic pain. Participants were suspected of having neuropathic pain if they had a score of ≥12 points on the Self-Reported Leeds Assessment of Neuropathic Symptoms and Signs pain scale (S-LANSS) [19, 20], which was included as part of the study questionnaire.

Secondary endpoints were the location of the pain, pain score based on an 11-point numeric rating scale (NRS; ranging from 0 = no pain to 10 = worst pain possible) [21], which healthcare professional the participant first consulted for pain (physician, nurse, pharmacist, or other healthcare professional), participant communication with healthcare professionals, impact of cancer pain on activities of daily living, types of analgesics used, QoL based on the EQ-5D-5L score [22], and the proportion of participants with S-LANSS ≥9.

### Statistical analysis

Considering feasibility for the distribution and processing of a paper survey, the maximum number of participants surveyed was set at 1000. Based on a previous study [23], it was assumed that the proportion of participants with suspected neuropathic pain would be between 5% and 15%. Assuming the number of participants with each type of cancer would be 100–140, the 95% confidence interval (CI) half-width was 3.6%–7.0%. The full analysis set (FAS) included all participants who responded to the questionnaire and had valid data available. The per-protocol set (PPS) included all participants in the FAS who indicated on the questionnaire that they had stage II (or higher) cancer.

Categorical variables were summarized using frequency and proportion, and continuous variables were summarized using descriptive statistics. For the primary endpoint, the proportion of participants with suspected neuropathic pain (S-LANSS score ≥ 12 points) and its 95% CI were calculated using the Clopper–Pearson method. Subgroup analyses were performed for populations with S-LANSS scores of ≥9 and ≥ 12 points.

Questionnaire items that had no response were categorized as “Unknown/no response.” If a participant selected multiple responses for a single-answer question, the response was included in the “unknown/no response” category. All statistical analyses were performed using SAS version 9.4 (SAS Institute Inc., Cary, NC, USA).

## Results

### Participants

**Figure 1:**
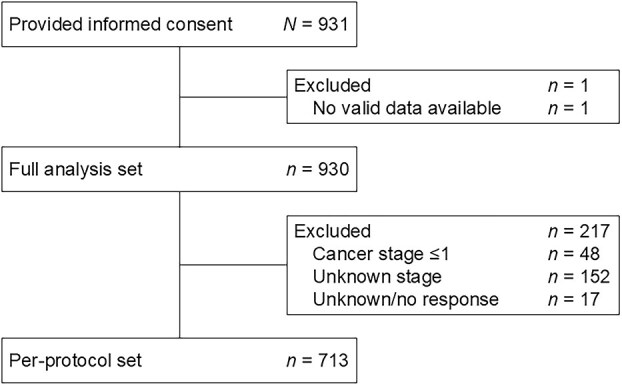
Patient disposition.

A total of 328 physicians from 134 institutions with 10 medical departments were asked to refer patients who were treated at those departments to this study, and paper questionnaires were distributed to 3789 patients. In total, 931 participants provided informed consent. The FAS included 930 participants (one was excluded due to a lack of valid data), and the PPS included 713 participants (217 were excluded from the FAS due to their current cancer stage response: Stage ≤I, *n* = 48; Don’t know, *n* = 152; Unknown/no response, *n* = 17) (Fig. 1). There were no duplicate respondents.

In the PPS, 371/713 (52.0%) participants were male; the most frequent age ranges were 60–69 years (193/713, 27.1%) and 70–79 years (262/713, 36.7%); the most frequent types of cancer were lung cancer (192/713, 26.9%), breast cancer (118/713, 16.5%), colorectal cancer (108/713, 15.1%), and other solid tumor (129/713, 18.1%), and 200/713 (28.1%), 200/713 (28.1%), and 313/713 (43.9%) had stage II, III, or IV cancer, respectively (Table 1). Most participants (503/713, 70.5%) were undergoing anticancer drug treatment regularly, and 461/713 (64.7%) had undergone surgery for their cancer. Most participants (551/713, 77.3%) did not have pain due to an injury or disease other than cancer.

**Table 1:** Participant characteristics in the overall population, the population with an S-LANSS score ≥ 12, and the population with an S-LANSS score ≥ 9 (per-protocol set).

	Overall (*N* = 713)	S-LANSS ≥12^a^ (*n* = 53)	S-LANSS ≥9^a^ (*n* = 66)
Q1: Sex			
Male	371 (52.0)	23 (43.4)	29 (43.9)
Female	338 (47.4)	30 (56.6)	37 (56.1)
No response	4 (0.6)	0 (0.0)	0 (0.0)
Q2: Age, years			
10 to <20	0 (0.0)	0 (0.0)	0 (0.0)
20 to <30	1 (0.1)	0 (0.0)	0 (0.0)
30 to <40	6 (0.8)	1 (1.9)	2 (3.0)
40 to <50	49 (6.9)	7 (13.2)	7 (10.6)
50 to <60	104 (14.6)	8 (15.1)	10 (15.2)
60 to <70	193 (27.1)	17 (32.1)	22 (33.3)
70 to <80	262 (36.7)	15 (28.3)	20 (30.3)
≥80	95 (13.3)	5 (9.4)	5 (7.6)
Unknown/no response	3 (0.4)	0 (0.0)	0 (0.0)
Q3: Type of cancer (multiple answers allowed)			
Stomach cancer	59 (8.3)	2 (3.8)	2 (3.0)
Lung cancer	192 (26.9)	4 (7.5)	9 (13.6)
Colorectal cancer	108 (15.1)	9 (17.0)	10 (15.2)
Breast cancer	118 (16.5)	17 (32.1)	20 (30.3)
Uterine/ovarian cancer	32 (4.5)	4 (7.5)	5 (7.6)
Prostate cancer	52 (7.3)	2 (3.8)	2 (3.0)
Other solid tumor	129 (18.1)	11 (20.8)	14 (21.2)
Blood cancer	1 (0.1)	0 (0.0)	0 (0.0)
Don’t know	2 (0.3)	0 (0.0)	0 (0.0)
Unknown/no response	20 (2.8)	4 (7.5)	4 (6.1)
Q4: Current cancer stage			
Stage 0	0 (0.0)	0 (0.0)	0 (0.0)
Stage I	0 (0.0)	0 (0.0)	0 (0.0)
Stage II	200 (28.1)	13 (24.5)	14 (21.2)
Stage III	200 (28.1)	17 (32.1)	23 (34.8)
Stage IV	313 (43.9)	23 (43.4)	29 (43.9)
Don’t know	0 (0.0)	0 (0.0)	0 (0.0)
Unknown/no response	0 (0.0)	0 (0.0)	0 (0.0)
Q5: Status of anticancer drug treatment			
Undergoing anticancer drug treatment regularly	503 (70.5)	38 (71.7)	49 (74.2)
Completed/discontinued previous anticancer treatment	137 (19.2)	11 (20.8)	12 (18.2)
Never received anticancer treatment	64 (9.0)	4 (7.5)	4 (6.1)
Don’t know	7 (1.0)	0 (0.0)	1 (1.5)
Unknown/no response	2 (0.3)	0 (0.0)	0 (0.0)
Q6: Surgery for cancer			
Have undergone surgery	461 (64.7)	38 (71.7)	48 (72.7)
Have not undergone surgery	249 (34.9)	15 (28.3)	18 (27.3)
Unknown/no response	3 (0.4)	0 (0.0)	0 (0.0)
Q7: Pain due to a disease other than cancer or an injury			
Present	126 (17.7)	15 (28.3)	20 (30.3)
Absent	551 (77.3)	36 (67.9)	44 (66.7)
Don’t know	34 (4.8)	2 (3.8)	2 (3.0)
Unknown/no response	2 (0.3)	0 (0.0)	0 (0.0)
Q8: Pain caused by cancer identified in Q3			
Yes	145 (20.3)	38 (71.7)	46 (69.7)
Maybe	85 (11.9)	15 (28.3)	20 (30.3)
No	476 (66.8)	0 (0.0)	0 (0.0)
Unknown/no response	7 (1.0)	0 (0.0)	0 (0.0)

Data are *n* (%).

Q, question; S-LANSS, Self-Reported Leeds Assessment of Neuropathic Symptoms and Signs.

^a^Includes participants who answered “Yes” or “Maybe” to Q8 of the study questionnaire.

Except for the male/female ratio, prevalence of breast and lung cancer, and pain-related characteristics, participant characteristics in the S-LANSS subgroups were similar to those in the overall PPS population (Table 1).

### Self-Reported Leeds Assessment of Neuropathic Symptoms and Signs pain scale score

**Figure 2:**
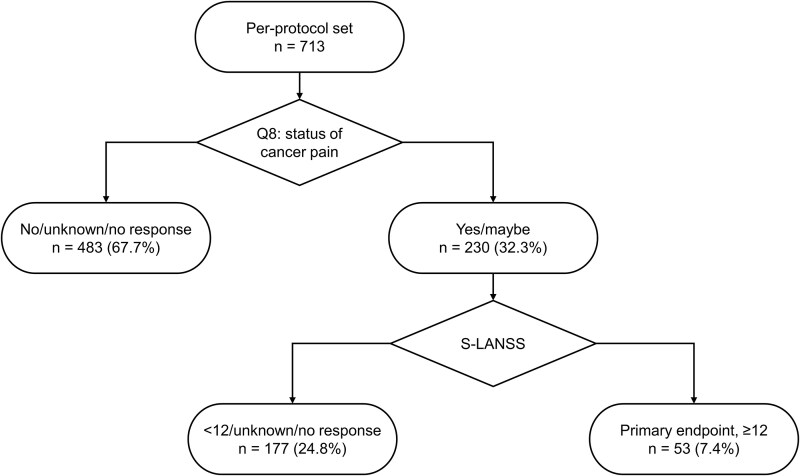
Flow diagram for Q8 of the study questionnaire, status of cancer pain. S-LANSS, Self-Reported Leeds Assessment of Neuropathic Symptoms and Signs.

**Figure 3:**
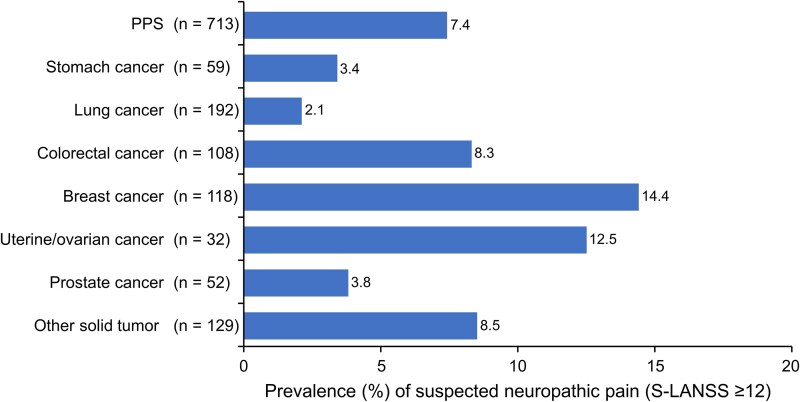
Prevalence of suspected neuropathic pain (S-LANSS ≥12) in the PPS and by cancer type. PPS, per-protocol set; S-LANSS, Self-Reported Leeds Assessment of Neuropathic Symptoms and Signs.

In the PPS, 230/713 participants (32.3%) answered “Yes” or “Maybe” to Q8 (pain caused by cancer) of the study questionnaire and 53/713 (7.4%) had an S-LANSS score ≥ 12 (Figs 2 and 3). By cancer type, an S-LANSS score ≥ 12 was most common among participants with breast cancer (14.4% [17/118]), uterine/ovarian cancer (12.5% [4/32]), other solid tumors (8.5% [11/129]), and colorectal cancer (8.3% [9/108]) (Fig. 3).

Among the 230 participants who answered “Yes” or ‘“Maybe” to Q8, 53/230 (23.0%) had a S-LANSS score ≥ 12, 81/230 (35.2%) reported experiencing tingling or prickling sensations such as “pins and needles,” 68/230 (29.6%) reported that the painful area was abnormally sensitive to touch, and 92/230 (40.0%) reported numbness and/or tenderness when they pressed the painful area (Table 2). Among participants with stage II, III, or IV cancer, the prevalence of S-LANSS scores ≥12 was similar: 6.5% (13/200), 8.5% (17/200), and 7.3% (23/313), respectively (Table 1).

**Table 2:** S-LANSS and pain-related outcomes in the overall population who answered “Yes” or “Maybe” to Q8 of the study questionnaire (per-protocol set).

	Overall (*n* = 230)^a^
S-LANSS Score ≥ 12 (primary endpoint)	53 (23.0)
Score < 12	172 (74.8)
Unknown/no response	5 (2.2)
Mean ± SD	n = 225 6.1 ± 6.9
Median (first quartile, third quartile)	3 (0, 11)
Min, max	0, 24
Q9-B-1: Do you also have tingling or prickling sensations such as “pins and needles” in the area where you have pain?	
No	148 (64.3)
Yes	81 (35.2)
Unknown/no response	1 (0.4)
Q9-B-2: When your pain is particularly bad, does the skin color on the area change (perhaps a mottled or an increased reddish appearance)?	
No	206 (89.6)
Yes	22 (9.6)
Unknown/no response	2 (0.9)
Q9-B-3: Does your pain make the area abnormally sensitive to touch? For instance, do you have something like unpleasant sensations or pain when the skin is gently stroked?	
No	160 (69.6)
Yes	68 (29.6)
Unknown/no response	2 (0.9)
Q9-B-4: Does your pain come on suddenly and in bursts for no apparent reason when you are completely still? Words like “electric shocks,” jumping, and bursting might describe this.	
No	192 (83.5)
Yes	37 (16.1)
Unknown/no response	1 (0.4)
Q9-B-5: Does the area where you have pain feel unusually hot like burning pain?	
No	205 (89.1)
Yes	23 (10.0)
Unknown/no response	2 (0.9)
Q9-B-6: Please gently rub the painful area with your index finger. Next, try to rub a nonpainful area in the same manner. For instance, try to do the same to an area quite far away or on the opposite side from the painful area. How does this rubbing feel in the painful area?	
No different from the nonpainful area	169 (73.5)
Discomfort	59 (25.7)
Unknown/no response	2 (0.9)
Q9-B-7: Please try to gently press on the painful area with your fingertip. Next, try to press on a nonpainful area in the same manner. Press on the same area that you chose in the preceding question. How does this feel in the painful area?	
No different from the nonpainful area	137 (59.6)
Numbness and/or tenderness	92 (40.0)
Unknown/no response	1 (0.4)

Data are *n* (%) unless otherwise specified.

Participants who answered “Yes” or “Maybe” to Q8 of the study questionnaire were directed to answer questions related to their pain in Q9-B-1 through Q9-B-7 (those who answered “No” skipped this set of questions).

Q, question; S-LANSS, Self-Reported Leeds Assessment of Neuropathic Symptoms and Signs.

^a^Includes participants who answered “Yes” or “Maybe” to Q8 of the study questionnaire.

### Pain characteristics and pain-related communication with healthcare professionals among participants with cancer pain who had suspected neuropathic pain (Self-Reported Leeds Assessment of Neuropathic Symptoms and Signs pain scale ≥ 12)

Among the 230 participants who answered “Yes” or “Maybe” to Q8, the mean ± standard deviation (SD) NRS score (pain intensity) over the week prior was 4.0 ± 2.4 (*n* = 222) (Table 3).

**Table 3:** Pain characteristics in the overall population who answered “Yes” or “Maybe” to Q8 of the study questionnaire and in subgroups of this population with an S-LANSS score ≥ 12 or an S-LANSS score ≥ 9 (per-protocol set).

	Overall (*N* = 230)^a^	S-LANSS ≥12 (*n* = 53)^a^	S-LANSS ≥9 (*n* = 66)^a^
Q9-A-1: Parts of the body that are most painful.			
Head	2 (0.9)	0 (0.0)	0 (0.0)
Face	1 (0.4)	1 (1.9)	1 (1.5)
Neck	7 (3.0)	1 (1.9)	1 (1.5)
Arm to hand	13 (5.7)	9 (17.0)	9 (13.6)
Shoulder	8 (3.5)	3 (5.7)	3 (4.5)
Chest	43 (18.7)	12 (22.6)	17 (25.8)
Back	15 (6.5)	2 (3.8)	3 (4.5)
Abdomen	78 (33.9)	9 (17.0)	12 (18.2)
Lower back	21 (9.1)	2 (3.8)	3 (4.5)
Buttocks	7 (3.0)	3 (5.7)	5 (7.6)
Legs to toe tips	19 (8.3)	8 (15.1)	9 (13.6)
Unknown/no response	16 (7.0)	3 (5.7)	3 (4.5)
Q9-A-2: Pain intensity^b^ over the last week in the areas where the pain is the worst.			
	*n* = 222	*n* = 50	*n* = 63
Mean ± SD	4.0 ± 2.4	5.1 ± 2.7	4.9 ± 2.7
Median (first quartile, third quartile)	3 (2, 6)	5 (3, 8)	5 (2, 7)
Q10: Did any healthcare professional (hospital staff) check with you about your cancer pain before you sought a consultation on your own?			
Yes	157 (68.3)	33 (62.3)	41 (62.1)
No	70 (30.4)	20 (37.7)	25 (37.9)
Unknown/no response	3 (1.3)	0 (0.0)	0 (0.0)
Q11: Who first checked with you about your cancer pain before you sought a consultation on your own?^c^			
	*n* = 157	*n* = 33	*n* = 41
Doctor	114 (72.6)	25 (75.8)	30 (73.2)
Nurse	16 (10.2)	4 (12.1)	5 (12.2)
Pharmacist	1 (0.6)	1 (3.0)	1 (2.4)
Other healthcare professional	3 (1.9)	0 (0.0)	0 (0.0)
Unknown/no response	23 (14.6)	3 (9.1)	5(12.2)
Q12: Have you, yourself, ever consulted with a healthcare professional about your pain?			
Yes	177 (77.0)	48 (90.6)	59 (89.4)
Never	45 (19.6)	4 (7.5)	5 (7.6)
Unknown/no response	8 (3.5)	1 (1.9)	2 (3.0)
Q13: Which healthcare professional did you consult first about your pain?^d^			
	*n* = 177	*n* = 48	*n* = 59
Doctor	137 (77.4)	40 (83.3)	50 (84.7)
Nurse	18 (10.2)	5 (10.4)	6 (10.2)
Pharmacist	2 (1.1)	2 (4.2)	2 (3.4)
Other healthcare professional	2 (1.1)	0 (0.0)	0 (0.0)
Unknown/no response	18 (10.2)	1 (2.1)	1 (1.7)
Q14: If you have not sought a consultation about pain, please choose a statement that best matches the reason.^e^			
	*n* = 45	*n* = 4	*n* = 5
It may be perceived as dissatisfaction with treatment	0 (0.0)	0 (0.0)	0 (0.0)
I have been prescribed pain medication	13 (28.9)	0 (0.0)	0 (0.0)
My pain is still bearable at present	21 (46.7)	2 (50.0)	3 (60.0)
I am concerned about increasing the number of drugs	1 (2.2)	0 (0.0)	0 (0.0)
The cancer treatment may be discontinued if I complain about pain	1 (2.2)	1 (25.0)	1 (20.0)
Other reasons	5 (11.1)	1 (25.0)	1 (20.0)
Unknown/no response	4 (8.9)	0 (0.0)	0 (0.0)
Q15: What intensity of pain would you tolerate without consulting a healthcare professional?^b^			
	*n* = 220	*n* = 52	*n* = 65
Mean ± SD	4.1 ± 2.3	3.7 ± 2.2	3.7 ± 2.2
Median (first quartile, third quartile)	4 (2, 5)	3 (2, 5)	3 (2, 5)
Q16: What is the degree of ease you feel about talking to a healthcare professional about pain?			
Doctor			
Easy	206 (89.6)	45 (84.9)	55 (83.3)
Difficult	20 (8.7)	7 (13.2)	9 (13.6)
Unknown/no response	4 (1.7)	1 (1.9)	2 (3.0)
Nurse			
Easy	205 (89.1)	45 (84.9)	56 (84.8)
Difficult	18 (7.8)	6 (11.3)	8 (12.1)
Unknown/no response	7 (3.0)	2 (3.8)	2 (3.0)
Pharmacist			
Easy	168 (73.0)	34 (64.2)	45 (68.2)
Difficult	46 (20.0)	16 (30.2)	17 (25.8)
Unknown/no response	16 (7.0)	3 (5.7)	4 (6.1)
Q17: Which healthcare professional is the easiest to talk to about pain?			
Doctor	142 (61.7)	36 (67.9)	43 (65.2)
Nurse	61 (26.5)	14 (26.4)	18 (27.3)
Pharmacist	15 (6.5)	2 (3.8)	4 (6.1)
Other healthcare professional	2 (0.9)	0 (0.0)	0 (0.0)
Unknown/no response	10 (4.3)	1 (1.9)	1 (1.5)
Q18: Do you wish that your healthcare professionals would notice your pain sooner?			
Yes	99 (43.0)	32 (60.4)	40 (60.6)
No	128 (55.7)	20 (37.7)	25 (37.9)
Unknown/no response	3 (1.3)	1 (1.9)	1 (1.5)
Q19: Are there any daily activities or routines that you can no longer do due to the pain of cancer?			
Yes	131 (57.0)	37 (69.8)	44 (66.7)
No	97 (42.2)	15 (28.3)	21 (31.8)
Unknown/no response	2 (0.9)	1 (1.9)	1 (1.5)
Q20: Is it necessary for the pain of cancer to go away to live your life going forward?			
Necessary	208 (90.4)	52 (98.1)	65 (98.5)
Unnecessary	19 (8.3)	0 (0.0)	0 (0.0)
Unknown/no response	3 (1.3)	1 (1.9)	1 (1.5)
Q21: What are the types of all the analgesics that you are using now?			
Opioid analgesics^f^	65 (9.1)	8 (15.1)	11 (16.7)
Pregabalin	18 (2.5)	3 (5.7)	4 (6.1)
Mirogabalin	34 (4.8)	6 (11.3)	8 (12.1)
Other analgesics^g^	175 (24.5)	27 (50.9)	35 (53.0)
Did not know the type	24 (3.4)	6 (11.3)	8 (12.1)
Did not use analgesics	434 (60.9)	16 (30.2)	16 (24.2)
Unknown/no response	30 (4.2)	1 (1.9)	3 (4.5)
Q22: Please give a response for each one of the analgesics you selected in Q21.^h^			
Opioid analgesics^f^	3.9 ± 1.1 (*n =* 65)	2.9 ± 1.5 (*n =* 8)	3.2 ± 1.4 (*n =* 11)
Pregabalin	3.9 ± 1.0 (*n =* 18)	3.7 ± 1.2 (*n =* 3)	3.8 ± 1.0 (*n =* 4)
Mirogabalin	3.2 ± 1.4 (*n =* 34)	2.8 ± 1.5 (*n =* 6)	2.6 ± 1.4 (*n =* 8)
Other analgesics^g^	3.9 ± 1.1 (*n =* 174)	3.0 ± 1.4 (*n =* 27)	3.3 ± 1.4 (*n =* 35)
Did not know the type of analgesics	3.5 ± 1.4 (*n =* 21)	2.7 ± 1.6 (*n =* 6)	2.8 ± 1.5 (*n =* 8)
Q23: What effects do you expect analgesics to provide?			
	*n* = 713^i^	*n* = 53	*n* = 66
Pain resolution	241 (33.8)	23 (43.4)	27 (40.9)
Pain alleviation with some residual pain	71 (10.0)	8 (15.1)	10 (15.2)
Improvement in pain-related interference with daily life	117 (16.4)	15 (28.3)	17 (25.8)
Enabling continuation with cancer treatment	46 (6.5)	5 (9.4)	8 (12.1)
Unknown/No response	238 (33.4)	2 (3.8)	4 (6.1)
Q24: Questionnaire to assess EQ-5D-5L			
	*n* = 686^i^	*n* = 52	*n* = 64
Mean ± SD	0.8204 ± 0.1788	0.6065 ± 0.2518	0.6060 ± 0.2436
Median (first quartile, third quartile)	0.8316 (0.7336, 1.0000)	0.6903 (0.4111, 0.8109)	0.6903 (0.4412, 0.7916)

Data are *n* (%) unless otherwise specified.

Q, question; S-LANSS, Self-Reported Leeds Assessment of Neuropathic Symptoms and Signs; SD, standard deviation.

^a^Includes participants who answered “Yes” or “Maybe” to Q8 of the study questionnaire.

^b^Measured using the numeric rating scale (range: 0 = no pain to 10 = worst pain possible).

^c^Includes participants who answered “Yes” to Q10 of the study questionnaire.

^d^Includes participants who answered “Yes” to Q12 of the study questionnaire.

^e^Includes participants who answered “No” to Q12 of the study questionnaire.

^f^Includes morphine, oxycodone, fentanyl, tramadol, or hydromorphone.

^g^Includes anti-inflammatory analgesics, acetaminophen, aspirin, sodium diclofenac, loxoprofen, or celecoxib.

^h^Mean ± SD of satisfaction with analgesics was assessed on a 5-point scale from 1 (dissatisfied) to 5 (satisfied).

^i^Number of responses obtained from the entire PPS group.

Among participants who answered “Yes” or “Maybe” to Q8 and had suspected neuropathic pain (S-LANSS ≥12) (*n* = 53), pain in the chest, abdomen, and arm to hand were most common (12/53 [22.6%], 9/53 [17.0%], and 9/53 [17.0%], respectively) (Q9-A-1) (Table 3). The mean ± SD NRS score (pain intensity) over the week prior was 5.1 ± 2.7 (*n* = 50) (Q9-A-2); 36.0% had mild pain (NRS 1–3), 28.0% had moderate pain (NRS 4–6), and 36.0% had severe pain (NRS 7–10) (data not shown). In the same population, 37.7% of participants (20/53) reported that no healthcare professional checked with them about their cancer pain before they sought a consultation about it themselves, while 62.3% (33/53) had been asked about pain (Q10). Most participants (90.6% [48/53]) had previously consulted with a healthcare professional about their pain (Q12). Of those, most participants had first consulted a physician (83.3% [40/48]) or a nurse (10.4% [5/48]) (Q13). Among the participants who did not consult a healthcare professional about their pain (7.5% [4/53]), the most common reason was that their pain was bearable (50.0% [2/4]), followed by concern that the cancer treatment might be discontinued if they complain and other reasons (25.0% [1/4] each) (Q14). Most participants (60.4% [32/53]) wished that their healthcare professional had noticed their pain sooner (Q18), and 69.8% of participants (37/53) reported that there were daily activities or routines that they could no longer do due to cancer pain (Q19). Almost all participants (98.1% [52/53]) thought that it was necessary for the pain of cancer to go away for them to live their life going forward (Q20).

### Pain medication among participants with cancer pain who had suspected neuropathic pain (Self-Reported Leeds Assessment of Neuropathic Symptoms and Signs pain scale ≥ 12)

Among participants who answered “Yes” or “Maybe” to Q8 and had suspected neuropathic pain (S-LANSS ≥12), 30.2% (16/53) reported that they did not use analgesics; 50.9% (27/53) used analgesics other than opioids, pregabalin, or mirogabalin; and 15.1% (8/53) used opioid analgesics (Table 3).

### Quality of life

**Figure 4:**
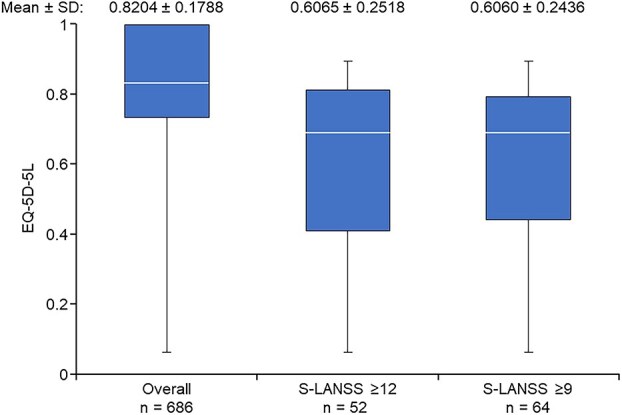
Quality of life based on EQ-5D-5L total score (overall population, S-LANSS ≥9, S-LANSS ≥12). The S-LANSS subgroup of participants included those who answered “Yes” or “Maybe” to Q8 of the study questionnaire. The line within the box represents the median, the bottom and top lines of the box represent the first and third quartiles, respectively, and the whiskers represent maximum or minimum. Q, question; SD, standard deviation; S-LANSS, Self-Reported Leeds Assessment of Neuropathic Symptoms and Signs.

In the PPS, the mean ± SD EQ-5D-5L total score was 0.8204 ± 0.1788; participants with S-LANSS scores of ≥12 (*n* = 52) and ≥ 9 (*n* = 64) had lower EQ-5D-5L scores than the PPS overall (0.6065 ± 0.2518 and 0.6060 ± 0.2436, respectively) (Fig. 4).

## Discussion

The study showed that 32.3% of participants experienced cancer-related pain and 7.4% had suspected neuropathic pain. Participants with suspected neuropathic pain reported a higher current pain intensity than that they considered tolerable. Both subgroups with S-LANSS scores ≥12 and ≥ 9 had a lower QoL compared with the overall population. Cancer-related pain prevented over half of the participants with suspected neuropathic pain from performing daily activities or routines, and many participants wanted relief from cancer-related pain. Many participants responded that they wished that their healthcare providers had noticed their pain earlier.

Although online survey methods are a tool that has been frequently used in recent studies of the same type [24], the target population of this study was patients with cancer and it was assumed that some of them would be older and unfamiliar with completing online questionnaires. Therefore, a paper survey was used to avoid age bias. To date, most patient surveys on cancer-related neuropathic pain have been conducted on a relatively small scale, either at a single institution or jointly by several institutions [25, 26], and there have been few large-scale cross-sectional studies spanning multiple departments and institutions. The distribution of cancer types in this study was generally consistent with the patient proportions in the Japanese Cancer Statistics Report [27]. Additionally, most existing reports have examined neuropathic pain as a subset of the population of patients with cancer-related pain. Our study started more broadly, screening all patients with cancer to identify those with cancer pain. Those patients were then further screened to identify patients with neuropathic pain. This three-step screening process makes our study unique from previous studies and provides useful information regarding the prevalence of neuropathic pain in the overall context of all cancer patients, not just those already identified as experiencing pain.

The purpose of this study was to examine the proportion of patients with suspected neuropathic pain, rather than patients definitively diagnosed with neuropathic pain. According to the Japanese Society of Pain Clinicians guideline [28], a definitive diagnosis of neuropathic pain requires a neurological examination by a physician and the use of a screening tool. In addition to the S-LANSS used in this study, other screening tools for neuropathic pain have been developed, including the Leeds Assessment of Neuropathic Symptoms and Signs (LANSS), the Douleur Neuropathique en 4 questions (DN4), painDETECT, the Neuropathic Pain Questionnaire (NPQ), and ID Pain questionnaires [29]. The S-LANSS was selected for use in the present study for two main reasons. First, there are numerous previous studies using the S-LANSS to assess neuropathic pain in patients with cancer [30–34]. Second, the questionnaire is self-administered and does not require the assistance of a medical professional.

This study focused on participants with S-LANSS scores ≥12, which has been established as a definitive indication of neuropathic pain [19], and also on participants with S-LANSS scores ≥9, which may be suggestive of neuropathic pain. Neuropathic pain can have various causes in patients with cancer, including the cancer itself, chemotherapy, and/or complications not directly related to the cancer. It was assumed that many patients with cancer have mixed pain that includes nociceptive, neuropathic, and central (nociplastic) pain. Considering that the S-LANSS was developed to identify pain of predominantly neuropathic origin [35], it may be less accurate for mixed pain than for neuropathic pain alone. In fact, several studies have reported a low sensitivity and/or specificity for tests developed to identify neuropathic pain when used in patients with cancer [23, 35, 36] or a reduced ability to detect neuropathic pain in patients with mixed pain [37]. Such issues may lead to a discrepancy between the pain assessment score and the patient’s own perception of their pain severity. Considering this, participants with S-LANSS scores ≥9 were investigated in addition to those with scores ≥12, and the findings were broadly comparable. Approximately 80% of patients with an S-LANSS score ≥9 also had a score ≥12. Participants with S-LANSS scores ≥12 and those with scores ≥9 had higher NRS scores for pain and lower QoL scores than the overall PPS population. These results suggest that an S-LANSS cutoff score of ≥12 may not be the only appropriate starting point for treatment of neuropathic pain in patients with cancer. Even if the S-LANSS score is lower (i.e. ≥9), treatment that plans for the possibility of neuropathic pain may be considered depending on their condition, which may be beneficial for patients.

This study reported the data from the PPS rather than the FAS, excluding the 217 participants with cancer stage ≤I or unknown cancer stage. Although a cancer stage of at least II was a prespecified inclusion criterion for this study, 48 and 169 participants in the FAS population had cancer stage ≤I and unknown cancer stage, respectively. While the attending physician confirmed each participant’s cancer stage prior to distributing the questionnaire, it is possible that some participants were not aware of their cancer stage.

In the present study, 32.3% of participants experienced cancer-related pain and 7.4% (23.0% of participants with cancer-related pain) had suspected neuropathic pain. This was generally consistent with a previous study [23]. Participants with suspected neuropathic pain (S-LANSS score ≥ 12) had greater pain severity than the overall PPS population (mean NRS score, 5.1 vs 4.0). The pain intensity in participants with S-LANSS scores ≥12 was greater than that considered by participants as tolerable pain (mean NRS score, 5.1 vs 3.7). Additionally, QoL scores (based on the EQ-5D-5L) were lower in participants with S-LANSS scores ≥12 versus the overall PPS population (0.6065 vs 0.8204). More participants with S-LANSS scores ≥12 consulted with a healthcare professional about their pain and wished that their healthcare professional had noticed their pain sooner, compared with the overall PPS population. These results suggest that participants with suspected neuropathic pain had more severe pain and more frequently consulted with a healthcare professional about their pain, but that they may have been experiencing pain of a greater-than-tolerable intensity when they first consulted a healthcare professional. Further, these patients experienced a diminished QoL. Compared with the overall PPS population, a consistently greater proportion of participants with suspected neuropathic pain reported that they could no longer perform daily activities or routines due to cancer pain (69.8% vs 57.0%) and that they thought it necessary for the pain of cancer to go away for them to live their life going forward (98.1% vs 90.4%). Taken together, these data suggest that healthcare professionals should conduct pain assessments at an earlier stage of cancer therapy and implement early intervention, rather than initiating treatment for pain after a patient voices that they are experiencing pain. As a new approach to pain assessment, it may be useful for earlier pain assessment to replace the traditional approach of healthcare providers asking patients “Do you have pain? How much pain do you feel?” with an approach based on the assumption that patients endure pain, such as “Do you have any pain that you think you can tolerate, but that is not severe or frequent enough to report at this time?”

This study had several limitations that should be considered when interpreting the findings. First, this was a survey study that collected responses provided by participants and was not based on objective evaluations by a third party such as a physician. To exclude responses from patients with noncancer neuropathic pain, the S-LANSS survey was not administered to patients who indicated that they had no pain from cancer. This study did not incorporate a definitive diagnosis of neuropathic pain per a physician-conducted neurological test. The reported prevalence of neuropathic pain was based on the S-LANSS screening tool, which has not been fully validated for patients with cancer pain. This study excluded patients with cancer stage 0 or I. This study was exploratory rather than confirmatory in nature; thus, statistical testing was not conducted. The number of cases with suspected neuropathic pain was small, and analysis by cancer type could not be performed in this population. Due to the nature of paper surveys, responses to some questions were not collected because they were unknown to the participant, or the answer was left blank. Finally, it would have been informative to investigate patient background according to pain medication, to understand which cancer types were associated with stronger pain medications. However, this study is limited to data collected at the time of the questionnaire survey, and data such as the duration of treatment and dosage of pain medications and concomitant medication were not collected. Therefore, it was difficult to examine the effects of pain medication based on the results of this study. Future studies should include comprehensive surveys of patients as well as medical professionals such as physicians and pharmacists.

## Conclusions

This study found that among participants with stage ≥II cancer, 32.3% had cancer pain and 7.4% had possible neuropathic pain. Participants with possible neuropathic pain had a lower QoL compared with the overall population and reported experiencing pain intensity that was greater than they could tolerate. These participants were more likely to wish that their healthcare providers had been aware of their pain earlier, before the participants themselves raised the issue. These findings suggest that starting pain assessment earlier during cancer treatment may help meet the potential needs of patients who are experiencing pain and want to consult with a healthcare provider about treating it.

## Supplementary Material

Supplementary_Material-rev_hyaf116

## References

[1] Bennett MI, Kaasa S, Barke A., et al. IASP taskforce for the classification of chronic pain. The IASP classification of chronic pain for ICD-11: Chronic cancer-related pain. Pain 2019;160:38–44. 10.1097/j.pain.0000000000001363. 30586069

[2] van den Beuken-van Everdingen MH, Hochstenbach LM, Joosten EA., et al. Update on prevalence of pain in patients with cancer: Systematic review and meta-analysis. J Pain Symptom Manag 2016;51:1070–90.e9. 10.1016/j.jpainsymman.2015.12.340. 27112310

[3] Neufeld NJ, Elnahal SM, Alvarez RH. Cancer pain: A review of epidemiology, clinical quality and value impact. Future Oncol 2017;13:833–41. 10.2217/fon-2016-0423. 27875910

[4] Prutianu I, Alexa-Stratulat T, Cristea EO., et al. Oxaliplatin-induced neuropathy and Colo-rectal cancer patient’s quality of life: Practical lessons from a prospective cross-sectional, real-world study. World J Clin Cases 2022;10:3101–12. 10.12998/wjcc.v10.i10.3101. 35647128 PMC9082707

[5] Roberto A, Deandrea S, Greco MT., et al. Prevalence of neuropathic pain in cancer patients: Pooled estimates from a systematic review of published literature and results from a survey conducted in 50 Italian palliative care centers. J Pain Symptom Manag 2016;51:1091–102.e4. 10.1016/j.jpainsymman.2015.12.336. 27017920

[6] Knoerl R, Mazzola E, Hong F., et al. Self-reported severity, characteristics, and functional limitations of chemotherapy-induced peripheral neuropathy. Pain Manag Nurs 2022;23:532–40. 10.1016/j.pmn.2021.11.010. 34972658

[7] Hirayama Y, Yoshida Y, Mori M, Tamura K. Effects of the publication of clinical guidelines for the management of chemotherapy-induced peripheral neuropathy on the administration preferences of oncology specialists: Japanese Association of Supportive Care in Cancer. Jpn J Clin Oncol 2020;50:897–902. 10.1093/jjco/hyaa056. 32424420

[8] Attal N, Cruccu G, Baron R., et al. EFNS guidelines on the pharmacological treatment of neuropathic pain: 2010 revision. Eur J Neurol 2010;17:1113–e88. 10.1111/j.1468-1331.2010.02999.x. 20402746

[9] Piano V, Schalkwijk A, Burgers J., et al. Guidelines for neuropathic pain management in patients with cancer: A European survey and comparison. Pain Pract 2013;13:349–57. 10.1111/j.1533-2500.2012.00602.x. 23067004

[10] Piano V, Verhagen S, Schalkwijk A., et al. Treatment for neuropathic pain in patients with cancer: Comparative analysis of recommendations in national clinical practice guidelines from European countries. Pain Pract 2014;14:1–7. 10.1111/papr.12036. 23360414

[11] Cruccu G, Truini A. A review of neuropathic pain: From guidelines to clinical practice. Pain Ther 2017;6:35–42. 10.1007/s40122-017-0087-0. 29178033 PMC5701894

[12] Swarm RA, Paice JA, Anghelescu DL., et al. Adult cancer pain, version 3.2019, NCCN clinical practice guidelines in oncology. J Natl Compr Cancer Netw 2019;17:977–1007. 10.6004/jnccn.2019.0038. 31390582

[13] Song SY, Ko YB, Kim H., et al. Effect of serotonin-norepinephrine reuptake inhibitors for patients with chemotherapy-induced painful peripheral neuropathy: A meta-analysis. Medicine (Baltimore) 2020;99:e18653. 10.1097/MD.0000000000018653. 31895829 PMC6946453

[14] German Guideline Program in Oncology. *S3-guideline. Palliative care for patients with incurable cancer. Short version 2.2. 2020*. https://www.leitlinienprogramm-onkologie.de/fileadmin/user_upload/Downloads/Leitlinien/Palliativmedizin/Version_2/GGPO_Palliative_Care_ShortVersion_2.2.pdf. (5 March 2025, date last accessed).

[15] Dellemijn P . Are opioids effective in relieving neuropathic pain? Pain 1999;80:453–62. 10.1016/S0304-3959(98)00256-5. 10342407

[16] Sun VC, Borneman T, Ferrell B., et al. Overcoming barriers to cancer pain management: An institutional change model. J Pain Symptom Manag 2007;34:359–69. 10.1016/j.jpainsymman.2006.12.011. PMC2747495 17616336

[17] Japan Ministry of Heath, Labour and Welfare . Ethical guidelines for medical and health research involving human subjects 2015. https://www.mhlw.go.jp/file/06-Seisakujouhou-10600000-Daijinkanboukouseikagakuka/0000080278.pdf (5 March 2025, date last accessed).

[18] Snijders RAH, Brom L, Theunissen M, van den Beuken-van Everdingen MHJ. Update on prevalence of pain in patients with cancer 2022: A systematic literature review and meta-analysis. Cancers (Basel) 2023;15:591. 10.3390/cancers15030591. 36765547 PMC9913127

[19] Bennett MI, Smith BH, Torrance N, Potter J. The S-LANSS score for identifying pain of predominantly neuropathic origin: Validation for use in clinical and postal research. J Pain 2005;6:149–58. 10.1016/j.jpain.2004.11.007. 15772908

[20] Bennett M . The LANSS pain scale: The Leeds assessment of neuropathic symptoms and signs. Pain 2001;92:147–57. 10.1016/s0304-3959(00)00482-6. 11323136

[21] Breivik H, Borchgrevink PC, Allen SM., et al. Assessment of pain. Br J Anaesth 2008;101:17–24. 10.1093/bja/aen103. 18487245

[22] EuroQol Group . *EQ-5D instruments: EQ-5D-5L*. https://euroqol.org/eq-5d-instruments/eq-5d-5l-about/ (5 March 2025, date last accessed).

[23] García de Paredes ML, Del Moral GF, Martínez Del Prado P., et al. First evidence of oncologic neuropathic pain prevalence after screening 8615 cancer patients. Results of the on study. Ann Oncol 2011;22:924–30. 10.1093/annonc/mdq449. 20926548

[24] Story DA, Tait AR. Survey research. Anesthesiology 2019;130:192–202. 10.1097/ALN.0000000000002436. 30688782

[25] Shabangu N, Thebe T, Casey M., et al. Chronic pain in female breast cancer survivors - prevalence, characteristics and contributing factors: A cross-sectional pilot study. BMC Womens Health 2023;23:613. 10.1186/s12905-023-02766-6. 37974174 PMC10655434

[26] Divella M, Vetrugno L, Bertozzi S., et al. Patient-reported pain and other symptoms among breast cancer survivors: Prevalence and risk factors. Tumori 2020;106:480–90. 10.1177/0300891620908930. 32162594

[27] Foundation for Promotion of Cancer Research . Cancer Statistics in Japan - 2024. https://ganjoho.jp/public/qa_links/report/statistics/pdf/cancer_statistics_2024_fig_E.pdf (19 March 2025, date last accessed).

[28] Sumitani M, Sakai T, Matsuda Y., et al. Executive summary of the clinical guidelines of pharmacotherapy for neuropathic pain: Second edition by the Japanese Society of Pain Clinicians. J Anesth 2018;32:463–78. 10.1007/s00540-018-2501-0. 29737410 PMC5973958

[29] Cruccu G, Truini A. Tools for assessing neuropathic pain. PLoS Med 2009;6:e1000045. 10.1371/journal.pmed.1000045. 19360134 PMC2661248

[30] Potter J, Higginson IJ, Scadding JW, Quigley C. Identifying neuropathic pain in patients with head and neck cancer: Use of the Leeds assessment of neuropathic symptoms and signs scale. J R Soc Med 2003;96:379–83. 10.1177/014107680309600804. 12893852 PMC539565

[31] Rojo RD, Ren JL, Lipe DN., et al. Neuropathic pain prevalence and risk factors in head and neck cancer survivors. Head Neck 2022;44:2820–33. 10.1002/hed.27199. 36129114

[32] Satija A, Joad AK, Rana SPS, Bhatnagar S. The burden of cancer-related neuropathic pain: A multi-centric cross-sectional observational study from North India. Indian J Palliat Care 2021;27:104–8. 10.4103/IJPC.IJPC_277_20. 34035626 PMC8121242

[33] Kerba M, Wu JS, Duan Q., et al. Neuropathic pain features in patients with bone metastases referred for palliative radiotherapy. J Clin Oncol 2010;28:4892–7. 10.1200/JCO.2010.28.6559. 20921451

[34] Hammond EA, Pitz M, Lambert P, Shay B. Quantitative sensory profiles of upper extremity chemotherapy induced peripheral neuropathy: Are there differences in sensory profiles for neuropathic versus nociceptive pain? Can J Pain 2019;3:169–77. 10.1080/24740527.2019.1665992. 35005406 PMC8730657

[35] Rayment C, Hjermstad MJ, Aass N., et al. European palliative care research collaborative (EPCRC). Neuropathic cancer pain: Prevalence, severity, analgesics and impact from the European palliative care research collaborative-computerised symptom assessment study. Palliat Med 2013;27:714–21. 10.1177/0269216312464408. 23175513

[36] Mercadante S, Gebbia V, David F., et al. Tools for identifying cancer pain of predominantly neuropathic origin and opioid responsiveness in cancer patients. J Pain 2009;10:594–600. 10.1016/j.jpain.2008.12.002. 19231297

[37] Higashibata T, Tagami K, Miura T., et al. Usefulness of painDETECT and S-LANSS in identifying the neuropathic component of mixed pain among patients with tumor-related cancer pain. Support Care Cancer 2020;28:279–85. 10.1007/s00520-019-04819-9. 31041583

