# Supplementary material for: A questionnaire-based cross-sectional study on neuropathic pain in patients with cancer in Japan
Source: Jpn J Clin Oncol. 2025 Aug 1;55(10):1141–51. doi: 10.1093/jjco/hyaf116 (PMC12501975; doi:10.1093/jjco/hyaf116)
Supplement: Supplementary_Material-rev_hyaf116 [file supplementary_material-rev_hyaf116.docx]

**Questionnaire on**

**Pain and Treatment in Cancer Patients**

***Items from the Self-Reported Leeds Assessment of Neuropathic Symptoms and Signs (S-LANSS) and EQ-5D-5L were used with permission from the respective copyright holders:

- Bennett MI, Smith BH, Torrance N, Potter J. The S-LANSS score for identifying pain of predominantly neuropathic origin: validation for use in clinical and postal research. J Pain 2005;6:149–58. <https://doi.org/10.1016/j.jpain.2004.11.007>.
- EuroQol Group. EQ-5D instruments: EQ-5D-5L. https://euroqol.org/eq-5d-instruments/eq-5d-5l-about/ (5 March 2025, date last accessed).

**◆Research Participation Consent**

**Please be sure to read the enclosed “Information about the Questionnaire Survey Research on Pain and Treatment in Cancer Patients.” If you would like to cooperate by participating in the survey, please check mark (✓) the box below.**

**After reading and understanding the information about this research, I voluntarily give consent to participate in this research.**

**◆Instructions for filling out the questionnaire**

- The questionnaire will take about 15 minutes to complete.
- Once you complete the answers, please place the questionnaire form in the enclosed return envelope and mail it without affixing postage no later than November 30, 2024 (Saturday).
- Since this is an anonymous survey, please **do not write any personal information such as your name or address** on the return envelope or the questionnaire form.
- **Please do not write anything other than your responses to the questionnaire on the response sheet.**
- In the survey, you will be asked about the drugs you use. If you don’t mind, please have **your medication record book ready**.

**First, we would like to ask questions about you.**

1. **What is your sex?**
   (Circle one)

| 1. Male | 1. Female |
| --- | --- |

1. **What is your age?**
   (Circle one)

| 1. 10s | 1. 20s | 1. 30s | 1. 40s |
| --- | --- | --- | --- |
| 1. 50s | 1. 60s | 1. 70s | 1. 80s or older |

1. **What is the type of cancer you have now?**

***If you have more than one, please choose the main type.**

(Circle one)

| 1. Stomach cancer | 1. Lung cancer | 1. Colorectal cancer |
| --- | --- | --- |
| 1. Breast cancer | 1. Uterine/ovarian cancer | 1. Prostate cancer |
| 1. Other solid tumor (cancer other than blood cancer) | 1. Blood cancer (e.g., leukemia, lymphoma, or multiple myeloma) | 1. Don’t know |

1. **What is the current stage of the cancer you identified in the response to Q3?**

(Circle one)

| 1. Stage 0 | 1. Stage I | 1. Stage II |
| --- | --- | --- |
| 1. Stage III | 1. Stage IV | 1. Don’t know |

1. **Which statement best describes the status of the anti-cancer drug treatment for the cancer you identified in the response to Q3?**

(Circle one)

| 1. I am **undergoing** anti-cancer drug treatment regularly | 1. I **have completed/discontinued** the anti-cancer drug treatment that I **underwent** |
| --- | --- |
| 1. I **have never undergone** any anti-cancer drug treatment | 1. Don’t know |

1. **Have you ever undergone surgery for the cancer you identified in the response to Q3?**
   (Circle one)

| 1. Yes, I **have undergone** surgery | 1. No, I **have not undergone** surgery |
| --- | --- |

1. **Are you having any pain due to a “disease other than cancer” or an “injury”?**
   (Circle one)

| 1. I **have** pain due to a disease other than cancer or an injury. | 1. I **do not have** pain due to a disease other than cancer or an injury. |
| --- | --- |
| 1. Don’t know |  |

**The next series of questions are about the pain you feel.**

1. **Does the cancer you identified in the response to Q3 cause any pain?**
   (Circle one)

| 1. Yes 2. Maybe 3. No **(→please proceed to Q21)** |
| --- |

**The framed questions below are for those who answered either “Yes” or “Maybe” to the question on whether cancer causes pain. Please answer Q9 through Q20 below about cancer pain.**

**9-A-1.**

- Please mark with a pencil or pen on the body diagram below where you feel the pain. If you have pain in more than one area, please only mark the one area where the pain is the worst.

Please select the body part closest to the “area where the pain is the worst” that you marked.
(Circle one)

| 1. Head | 1. Face | 1. Neck |
| --- | --- | --- |
| 1. Arm to hand | 1. Shoulder | 1. Chest |
| 1. Back | 1. Abdomen | 1. Lower back |
| 1. Buttocks | 1. Leg to toe tips |  |

**Please answer the question below about cancer pain.**

**9-A–2.**

- On a scale from zero to ten, please select the intensity of your pain over the last week in the area you selected in 9-A-1 as where the pain is the worst. Zero means no pain, and ten is the worst pain imaginable.

(Circle one **number**)

**No pain 0 1 2 3 4 5 6 7 8 9 10 Worst pain**

- On the next page, there are seven questions about your pain in the area you selected in 9-A-1 as where the pain is the worst.

How has your pain in the area you selected in 9-A-1 as where the pain is the worst felt **over the last week**? Please circle the description that best matches your pain. Some of the questions may not be directly related to the pain you felt.

- You may just circle the one that matches your pain. **Please proceed to the next page.**

**Please answer the following questions about your pain in the area you selected in 9-A-1 as where the pain is the worst.**

1. **Do you also have tingling or prickling sensations such as “pins and needles” in the area where you have pain?**

(Circle one)

| 1. No ― I do not have such sensations |
| --- |
| 1. Yes ― I have such sensations often |

1. **When your pain is particularly bad, does the skin color on the area change (perhaps a mottled or an increased reddish appearance)?**

(Circle one)

| 1. No ― The skin color does not change even when the pain is bad |
| --- |
| 1. Yes ― The skin color has at times appeared different than usual when I had pain |

1. **Does your pain make the area abnormally sensitive to touch? For instance, do you have something like unpleasant sensations or pain when the skin is gently stroked?**

(Circle one)

| 1. No ― The area does not become sensitive to touch |
| --- |
| 1. Yes ― The area would become very sensitive to touch |

1. **Have you ever developed pain that seems to erupt suddenly for no apparent reason while staying still/not moving? It may feel as though you are getting “electric shocks,” your body is jumping up, and something inside your body is suddenly erupting violently.**

(Circle one)

| 1. No ― My pain is not like that |
| --- |
| 1. Yes ― I have such a sensation often |

**Please answer the following questions about your pain in the area you selected in 9-A-1 as where the pain is the worst.**

1. **Does the area where you have pain feel unusually hot like burning pain?**

(Circle one)

| 1. No ― I don’t have such pain |
| --- |
| 1. Yes ― I have such pain often |

1. **Please gently rub the painful area with your index finger. Next, try to rub a non-painful area in the same manner. For instance, try to do the same to an area quite far away or on the opposite side from the painful area. How does this rubbing feel in the painful area?**

(Circle one)

| 1. The painful area feels no different from the non-painful area |
| --- |
| 1. I feel discomfort in the painful area, like tingling or prickling sensations such as pins and needles or burning, which is different from that in the non-painful area |

1. **Please try to gently press on the painful area with your fingertip. Next, try to press on a non-painful area in the same manner. Press on the same area that you chose in the preceding question. How does this feel in the painful area?**

(Circle one)

| 1. The painful area feels no different from the non-painful area |
| --- |
| 1. I feel numbness and/or tenderness in the painful area, which is different from that in the non-painful area |

*Chiharu Usui: Validation of Self-Reported Leeds Assessment of Neuropathic Pain [Symptoms and Signs]* **[Please note that the underlined is included to explain the abbreviation that follows.]** *(S-LANSS) Associated with a Motor System Disease, Japanese version. The Journal of the Japanese Society for the Study of Chronic Pain 32(1): 233-236, 2013, partially revised*

**Please answer the questions below about cancer pain.**

1. **Did any healthcare professional (hospital staff) check with you about your cancer pain before you sought a consultation on your own?**

(Circle one)

| 1. Yes | 1. No |
| --- | --- |

The following question is for those who responded “Yes” to Q10.

1. **Who first checked with you about your cancer pain before you sought a consultation on your own?**
   (Circle one)

| 1. A doctor | 1. A nurse | 1. A pharmacist |
| --- | --- | --- |
| 1. Other healthcare professional |  |  |

**Please answer the questions below about cancer pain.**

1. **Have you, yourself, ever consulted with a healthcare professional about your pain?**
   (Circle one)

| 1. Yes | 1. No |
| --- | --- |

The following question is for those who responded “Yes” to Q12.

1. **Which healthcare professional did you consult first about your pain?**
   (Circle one)

| 1. A doctor | 1. A nurse | 1. A pharmacist |
| --- | --- | --- |
| 1. Other healthcare professional |  |  |

The following question is for those who responded “No” to Q12.

1. **If you have not sought a consultation about pain, please choose a statement that best matches the reason.**
   (Circle one)

| 1. It may be perceived as dissatisfaction with the treatment | 1. I have been prescribed pain medication | 1. My pain is still bearable at present |
| --- | --- | --- |
| 1. I am concerned about increasing the number of drugs | 1. The cancer treatment may be discontinued if I complain about pain | 1. Other reasons |

**Please answer the questions below about cancer pain.**

1. **What intensity of pain would you tolerate without consulting a healthcare professional? Please choose an intensity from zero to ten. Zero means no pain, and ten is the worst pain imaginable.**

***Please choose the worst pain you would tolerate without seeking a consultation.**
(Circle one **number**)

**No pain 0 1 2 3 4 5 6 7 8 9 10 Worst pain**

1. **What is the degree of ease you feel about talking to a healthcare professional about pain?**
   (Circle one)
2. A doctor

| 1. Easy | 1. Difficult |
| --- | --- |

1. A nurse

| 1. Easy | 1. Difficult |
| --- | --- |

1. A pharmacist

| 1. Easy | 1. Difficult |
| --- | --- |

**Please answer the questions below about cancer pain.**

1. **Which healthcare professional is the easiest to talk to about pain?**
   (Circle one)

| 1. A doctor | 1. A nurse | 1. A pharmacist |
| --- | --- | --- |
| 1. Others |  |  |

1. **Do you wish that your healthcare professionals would notice your pain sooner?**
   (Circle one)

| 1. Yes | 1. No |
| --- | --- |

1. **Are there any daily activities or routines that you can no longer do due to the pain of cancer?**
   (Circle one)

| 1. Yes | 1. No |
| --- | --- |

1. **Is it necessary for the pain of cancer to go away to live your life going forward?**
   (Circle one)

| 1. Yes | 1. No |
| --- | --- |

**The next series of questions are about analgesics (pain medication).**

1. **What are the types of all the analgesics that you are using now?**
   **(Circle all that apply)**

| 1. Opioid analgesics (e.g., morphine, oxycodone, fentanyl, or hydromorphone) | 1. Lyrica (pregabalin) |
| --- | --- |
| 1. Tarlige (mirogabalin) | 1. Other analgesics (anti-inflammatory analgesics or other analgesics such as acetaminophen, aspirin, sodium diclofenac, loxoprofen, or celecoxib) |
| 1. I am using an analgesic(s), but I don’t know the type(s). | 1. I am not using analgesics. |

**The following question is for those who responded to Q21 by choosing an answer between 1 and 5.**

1. **On a scale from one to five, how satisfied are you with your analgesics? One means dissatisfied, and five means satisfied.**

**Please give a response for each one of the analgesics you selected in Q21**.

1. **Opioid analgesics**

**Dissatisfied 1 2 3 4 5 Satisfied**

1. **Lyrica (pregabalin)**

**Dissatisfied 1 2 3 4 5 Satisfied**

1. **Tarlige (mirogabalin)**

**Dissatisfied 1 2 3 4 5 Satisfied**

1. **Other analgesics**

**Dissatisfied 1 2 3 4 5 Satisfied**

1. **The drug, the type of which I do not know**

**Dissatisfied 1 2 3 4 5 Satisfied**

1. **What effects do you expect analgesics to provide? Please choose one that best matches your expectation.**
   (Circle one)

| 1. Pain resolution | 1. Pain alleviation with some residual pain | 1. Improvement in pain-related interference with daily life |
| --- | --- | --- |
| 1. Enabling continuation with cancer treatment |  |  |


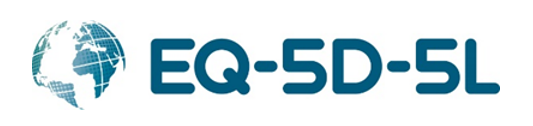


1. **Questionnaire to assess EQ-5D-5L.**
